# Successful Percutaneous Balloon Angioplasty in a Patient Presenting With STEMI and Acute Intracranial Hemorrhage

**DOI:** 10.7759/cureus.15166

**Published:** 2021-05-22

**Authors:** Aref Obagi, Matthew Schoenfeld

**Affiliations:** 1 Cardiology, Jersey Shore University Medical Center, Neptune, USA; 2 Interventional Cardiology, Jersey Shore University Medical Center, Neptune, USA

**Keywords:** st-elevation myocardial infarction (stemi), stemi, st-segment elevation myocardial infarction (stemi), balloon angioplasty, percutaneous transluminal balloon angioplasty, intracranial hemorrage

## Abstract

Percutaneous coronary interventions (PCI) mandates the administration of anti-platelet and anti-thrombotic agents to prevent intracoronary and post-procedural thrombosis upon introducing thrombogenic foreign bodies such as intracoronary wires, balloons, or stents, especially in the setting of acute coronary syndrome (ACS) given the hypercoagulable state associated with it. This is a case of a 54-year-old female who presented to the emergency department with left-sided weakness and dysarthria for an unknown duration. A CT scan of the head showed acute right middle cerebral artery distribution infarct. She subsequently underwent a successful thrombectomy. Four hours later, the patient became lethargic and nauseous. Electrocardiogram showed anterior wall ST elevation with new-onset anterior wall akinesia on transthoracic echocardiogram. Repeat CT of the head showed acute intracranial hemorrhagic conversion. She then developed cardiac arrest mandating emergent cardiac catheterization. Coronary angiogram revealed 100% occlusion in a mid left anterior descending artery (LAD) and 80% in a left circumflex artery (LCX) and chronic total occlusion of the right coronary artery (RCA). After weighing risks and benefits, PCI was performed with rapid plain old balloon angioplasty (POBA) to the 100% thrombotic lesion in the LAD with successful restoration of flow without administering anti-platelet or anti-thrombotic agents given the acute intracranial hemorrhage, She was then discharged to a rehab facility a few days later in stable condition. This case demonstrates successful percutaneous coronary intervention in the 100% occluded LAD in a life-threatening situation despite not using anticoagulation or antiplatelet therapy due to active intracranial hemorrhage.

## Introduction

An acute ST-elevation myocardial infarction (STEMI) is an event in which transmural myocardial ischemia results in myocardial necrosis or injury [1]. A minority of patients who sustain an acute ST-elevation myocardial infarction do so while hospitalized for another reason (similar to our case). Patients developing STEMI after hospital admission have different baseline characteristics and have worse outcomes than those who present to the emergency room [2]. Initial management for such patients relies heavily on antiplatelet and antithrombic therapies prior to revascularization with strong supporting evidence. We present a unique case in which we were able to perform percutaneous coronary revascularization without initiating these therapies due to absolute contraindication because of the intracranial hemorrhage.

## Case presentation

**Figure 1:**
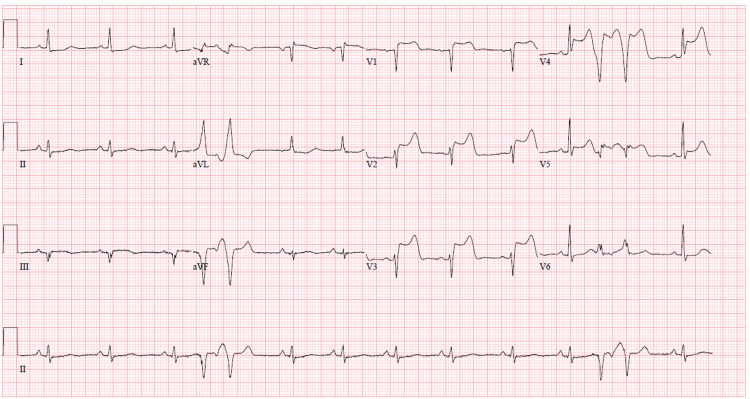
EKG showing ST elevations in V1 through V5 along with premature ventricular complexes.

A 54-year-old female with a history of hypertension and tobacco use presented with left-sided weakness and dysarthria for unknown duration. CT scan of the head showed acute right middle cerebral artery (MCA) distribution ischemic infarct. The patient was not a candidate for fibrinolytic therapy due to unknown duration of symptoms and she was transferred to our facility for thrombectomy which was successfully performed. She was transferred to the intensive care unit in stable condition. Shortly after this, she complained of nausea and chest discomfort. 12-lead electrocardiogram demonstrated acute anterior STEMI (Figure 1). Bedside echocardiogram showed hypokinesia of the entire apex (Video 1).

**Video 1:** Transthoracic echocardiogram with apical four chamber view showing apical wall akinesia.

**Figure 2:**
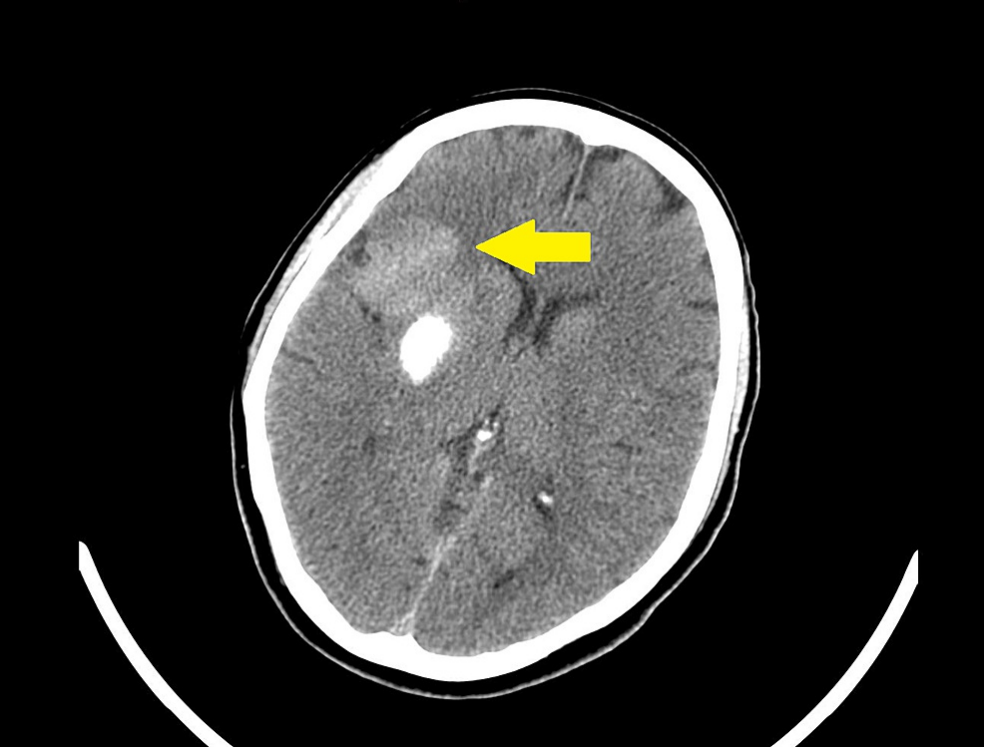
CT of the head showing hemorrhagic conversion in the basal ganglia (yellow arrow). CT: computed tomography.

Emergent CT of the head was performed prior to possible PCI and revealed hemorrhagic conversion in the basal ganglia (Figure 2). Because of the new hemorrhagic conversion, we decided not to intervene with close monitoring in the intensive care unit.

**Figure 3:**
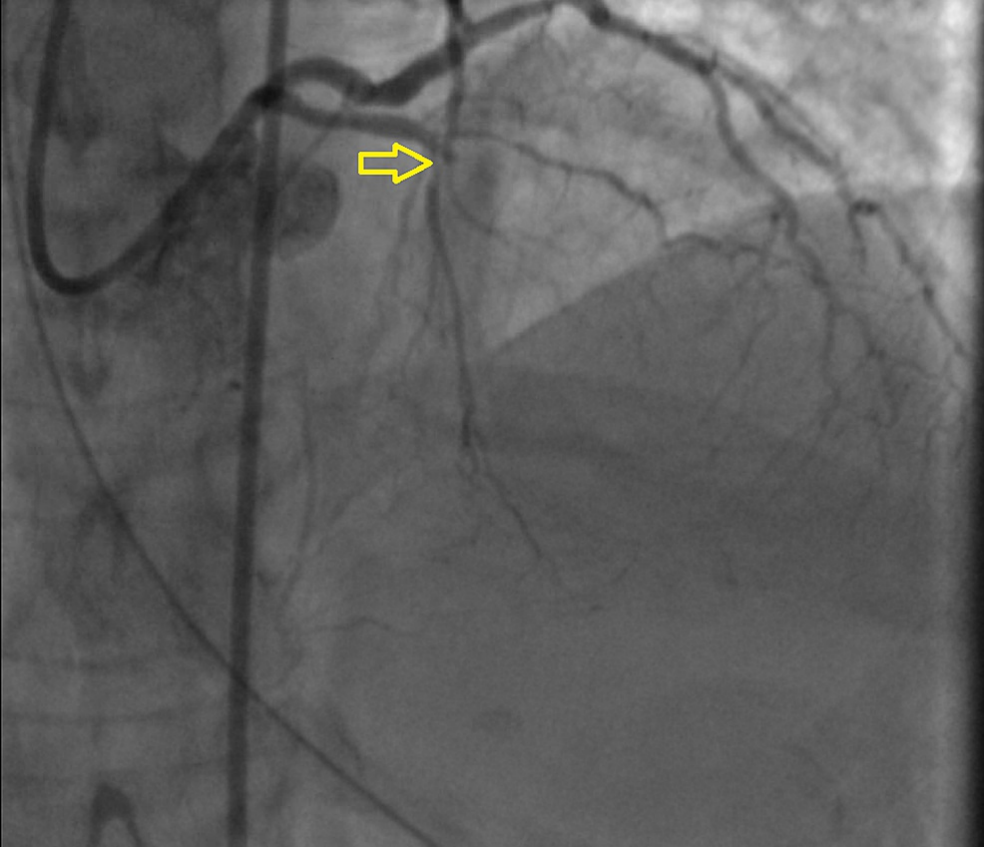
100% occlusion in mid LAD artery (yellow arrow). LAD: left anterior descending.

**Figure 4:**
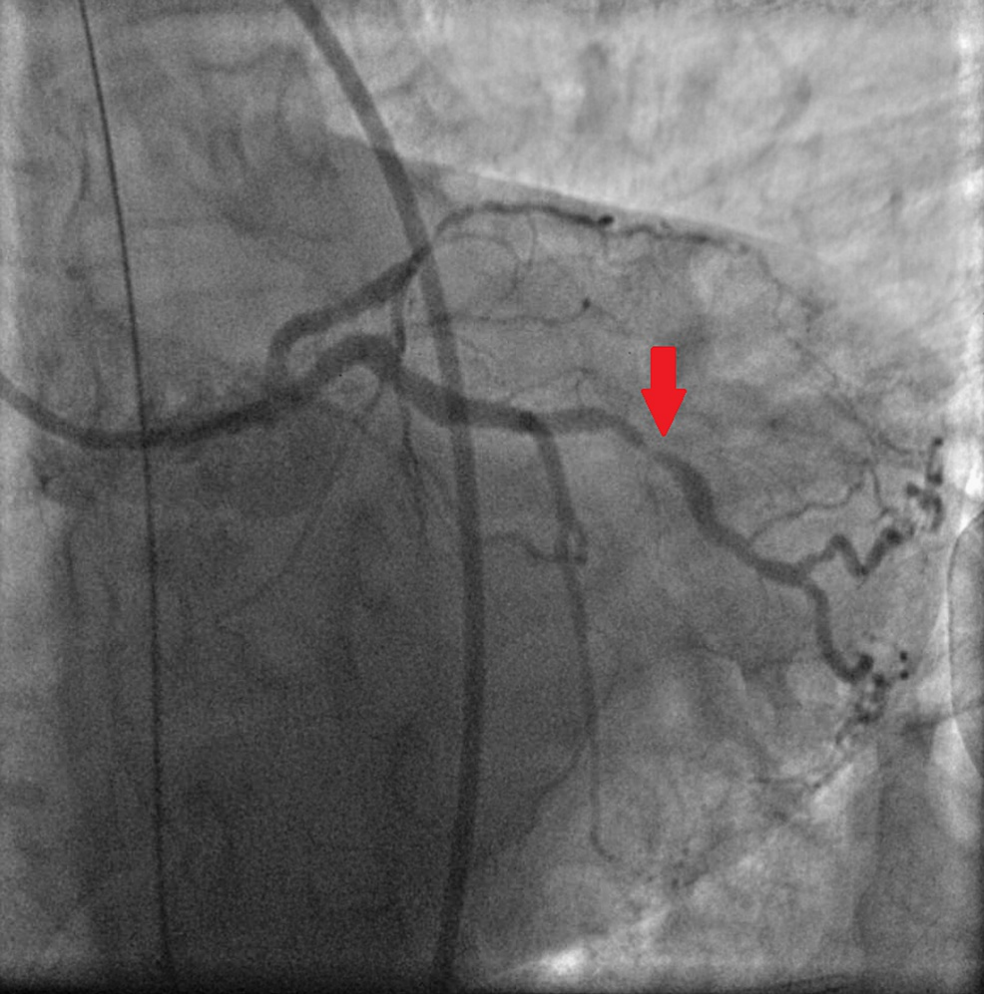
80% stenosis in a large obtuse marginal branch (red arrow).

**Figure 5:**
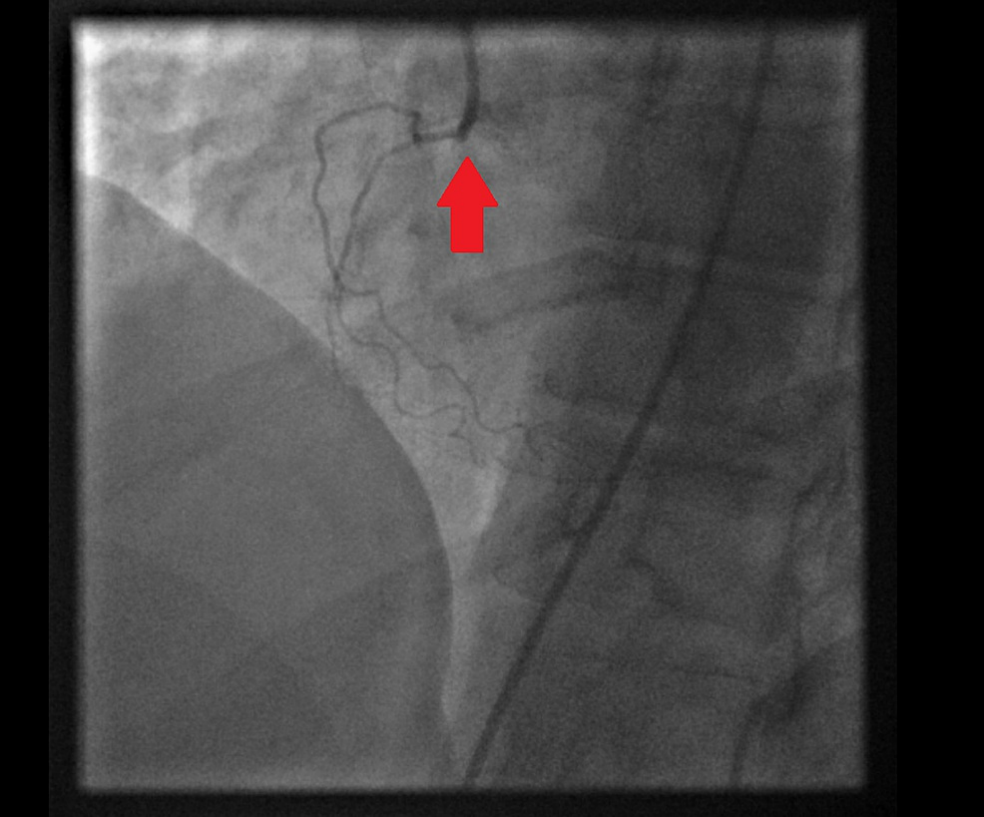
Chronic total occlusion of right coronary artery (red arrow).

Forty-five minutes later, the patient developed polymorphic ventricular tachycardia requiring cardiopulmonary resuscitation (CPR) and electrical cardioversion. Return of spontaneous circulation (ROSC) was achieved and amiodarone was initiated. Given these findings the decision was made to proceed with emergent coronary angiogram with plans for percutaneous transluminal coronary angioplasty (PTCA) without the use of antithrombotic or antiplatelet therapy. Coronary angiography via femoral approach revealed thrombotic occlusion of the mid left anterior descending (LAD) artery (Figure 3), 80% stenosis in a large obtuse marginal branch (Figure 4) and chronic total occlusion of right coronary artery (RCA) (Figure 5).

 

 

**Figure 6:**
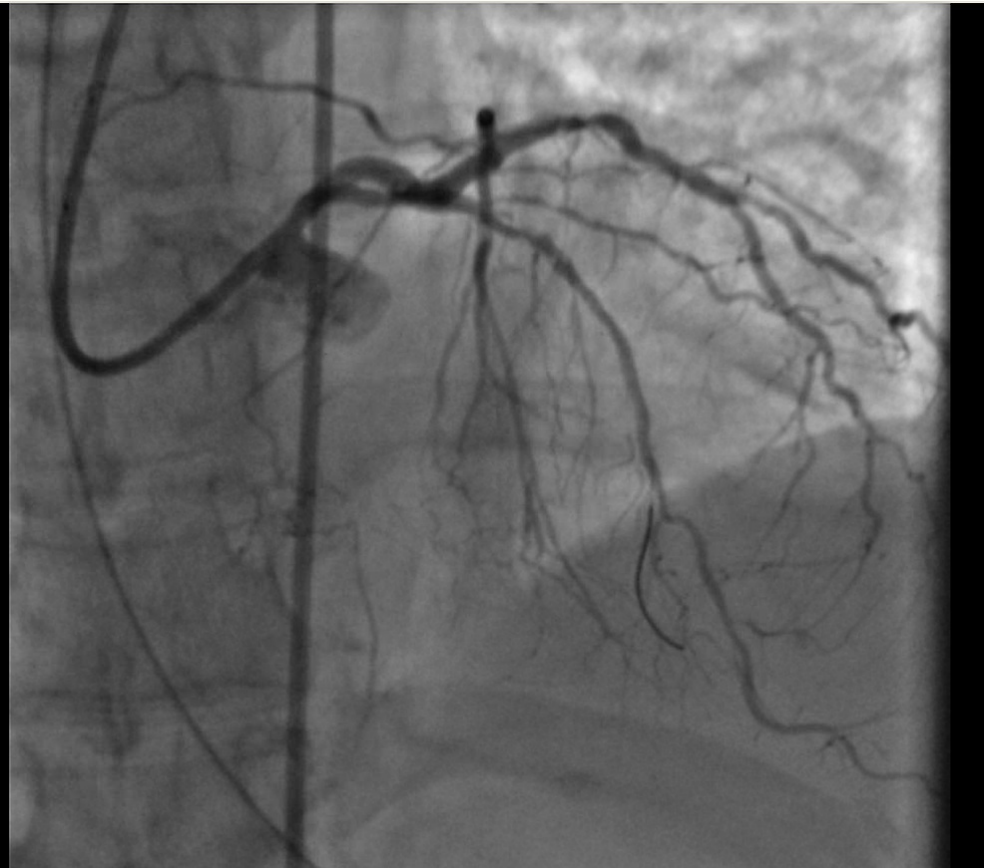
LAD after PTCA using 2.0 x 15 mm compliant balloon. LAD: left anterior descending; PTCA: percutaneous transluminal coronary angioplasty.

Percutaneous coronary intervention (PCI) with plain old balloon angioplasty (POBA) was rapidly performed using 2.0 x 15 mm compliant balloon with resultant TIMI III flow (Figure 6).

**Figure 7:**
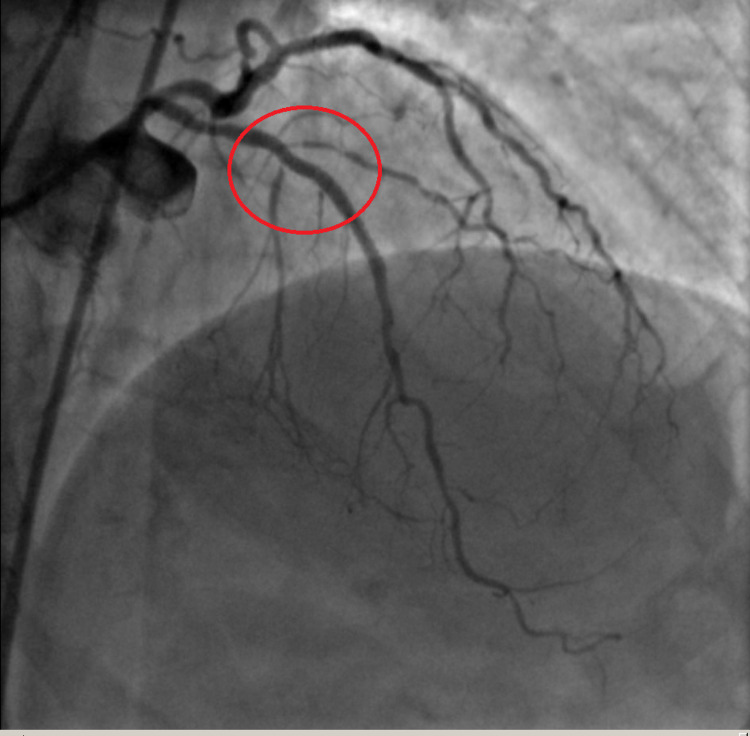
LAD artery after stent placement (red circle). LAD: left anterior descending.

**Figure 8:**
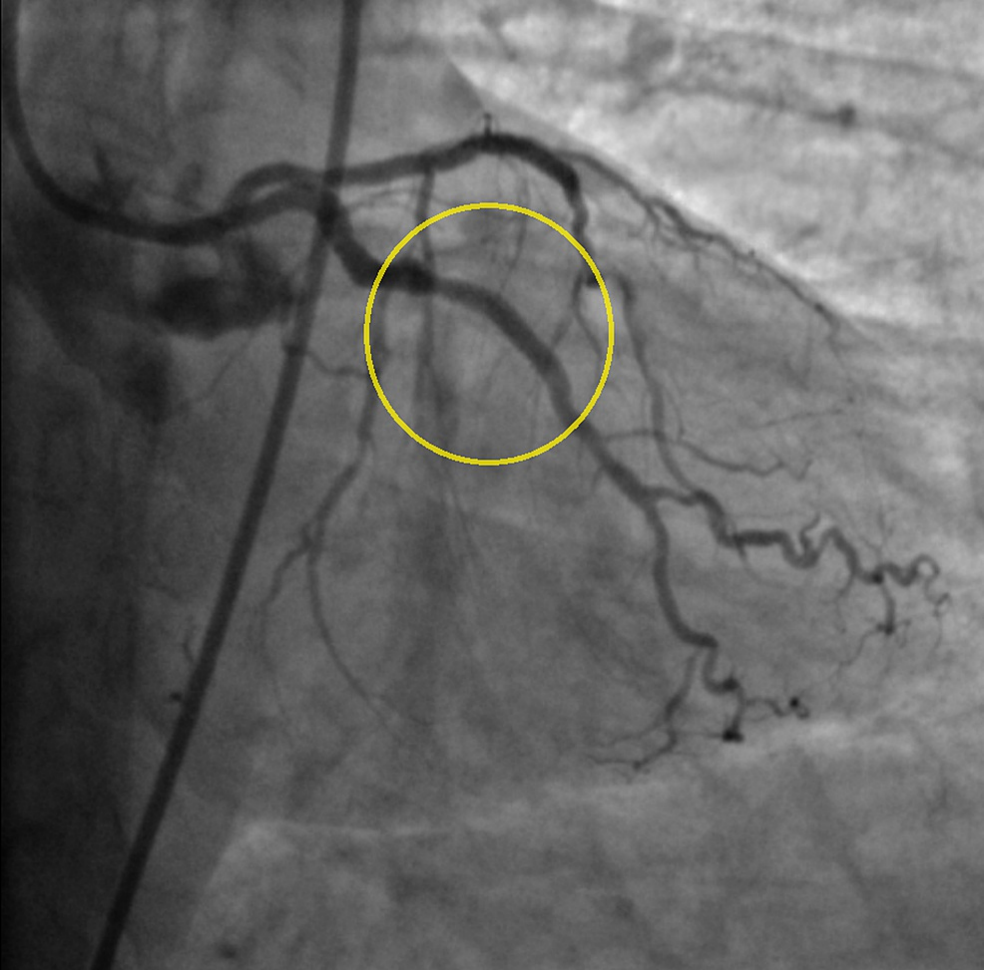
Obtuse marginal after stent placemen (yellow circle).

Two days later the patient was started on aspirin 81 mg daily. She was discharged in stable condition with significant improvements in her neurologic status and minimal residual upper extremity weakness. Once cleared for clopidogrel therapy and procedural anticoagulation she underwent elective coronary angiogram which demonstrated TIMI III flow in the LAD with residual stenosis. Successful PCI of the LAD (Figure 7) and obtuse marginal (Figure 8) was performed with two drug-eluting stents.

 

A two-month follow-up transthoracic echocardiogram showed improvement in left ventricle ejection fraction. 

## Discussion

ST-elevations can occur in up to 12% of patients with intracerebral hemorrhage and may be related to stress cardiomyopathy or rarely, concomitant STEMI [3]. Consideration of an embolic nature of coronary occlusion is important [3].

Antithrombotic and antiplatelet therapies are considered as class 1 indications for patients presenting with STEMI and undergoing PCI according to the 2017 European Society of Cardiology (ESC) guidelines [4]. Their usage prevents intra and post-procedural thrombosis especially since patients presenting with ACS are hypercoagulable and the use of intracoronary foreign bodies (such as intracoronary wires, balloons, and stents) predispose to thrombus formation [5]. 

Contraindication to their usage is generally viewed as an absolute contraindication to PCI. According to a double-blinded, randomized, prospective trial (CIAO trial), it compared standard anticoagulation regimen to the absence of anticoagulation for elective PCI along with the use of dual anti-platelet [6], however to our knowledge, there has not been a trial or case report of patients presenting with ACS (especially STEMI) and undergoing PCI without anticoagulation and/or antiplatelet therapy.

In a study comparing coronary stent placement vs balloon angioplasty, placement of an intracoronary stent resulted in a lower rate of angiographically detected restenosis, an improved rate of procedural success, a similar rate of clinical events after six months and a lower rate of angiographically detected restenosis [7]. Rate of restenosis in the balloon angioplasty arm estimated at 42.1% versus stent placement of 31.6% (P=0.046) [7].

Another study concluded that the re-stenosis rate in patients underwent balloon angioplasty was 32% versus 18% in patient underwent stent placement (P=0.03) [8].

Our case report demonstrated that the culprit vessel remained patent with TIMI III flow several weeks after plain old balloon angioplasty which was the only bailout option available at the time of the acute myocardial infarction.

## Conclusions

Management of ST-elevation myocardial infarction can be challenging and carries poor prognosis if its associated acute intracranial hemorrhage (ICH), since PCI requires pre or intra-procedural administration of anti-platelet and anti-thrombotic agents (to prevent intracoronary thrombus formation due to the hypercoagulable state associated with STEMI plus introducing foreign bodies such as wires, balloons, and stents) which are absolutely contraindicated in the setting of ICH. Here, we report a case of a young, previously healthy patient who underwent PCI with rapid plain old balloon angioplasty (POBA) without administration of antithrombotic or antiplatelet agents as a bailout to restore flow to a 100% occluded LAD after developing cardiac arrest and intracranial hemorrhage with good outcomes. 
